# 

**Published:** 1862-02

**Authors:** 


### BOOKS RECEIVED.

*A System of Surgery; Pathological, Diagnostic, Therapeutic and Operative, by* SAMUEL D. GROSS, M. D., *Professor of Surgery in the Jefferson Medical College of Philadelphia; Surgeon to the Philadelphia Hospital; Member of the Imperial Royal Medical Society of Vienna' etc., etc. Illustrated by twelve hundred and twenty-seven engravings. Second edition, much enlarged and carefully revised, in two volumes. Philadelphia:* BLANCHARD & LEA, 1862.

Received through, and for sale by, BREED, BUTLER & Co., No. 188 Main street, Buffalo.

---

*Notes on the Surgery of the War of the Crimea, with remarks on the treatment of gunshot wounds, by* GEORGE H. B. MACLEOD, M. D., F. R. C. S.; *formerly Surgeon to the Civil Hospital at Smyrna, and to the General Hospital in Camp before Sebastopol; Lecturer on Military Surgery in Anderson University, Glasgow. etc. Philadelphia:* J. B. LIPPINCOTT & Co., 1862. *London:* JOHN CHURCHILL.

---

*Harpers' New Monthly Magazine, for February,* 1862, *with its usual variety of history, incident and story. Illustrated with beautiful engravings.*

